# Effects of Antithrombin on Persistent Inflammation, Immunosuppression, and Catabolism Syndrome among Patients with Sepsis-Induced Disseminated Intravascular Coagulation

**DOI:** 10.3390/jcm12113822

**Published:** 2023-06-02

**Authors:** Naoki Kanda, Hiroyuki Ohbe, Kensuke Nakamura

**Affiliations:** 1 Department of Emergency and Critical Care Medicine, Hitachi General Hospital, 2-1-1 Jonan, Hitachi 317-0077, Ibaraki, Japan; 2 Division of General Internal Medicine, Jichi Medical University Hospital, 3311-1, Yakushiji, Shimotsuke 329-0431, Tochigi, Japan; 3 Department of Clinical Epidemiology and Health Economics, School of Public Health, The University of Tokyo, 7-3-1 Hongo, Bunkyo-ku, Tokyo 113-8654, Japan; 4 Department of Critical Care Medicine, Yokohama City University Hospital, 3-9 Fukuura, Kanazawa-ku, Yokohama 236-0004, Kanagawa, Japan

**Keywords:** PICS, persistent inflammation, DIC, coagulopathy, antithrombin

## Abstract

Persistent inflammation, immunosuppression, and catabolism syndrome (PICS) is a serious condition after critical care. We examined the efficacy of antithrombin, which may attenuate coagulopathy with the control of inflammation, for PICS among patients with sepsis-induced disseminated intravascular coagulation (DIC). The present study used the inpatient claims database with laboratory findings to identify patients admitted to intensive care units and diagnosed with sepsis and DIC. A composite of the incidence of PICS on day 14 or 14-day mortality as the primary outcome was compared between the antithrombin and control groups using a propensity-score-matched analysis. Secondary outcomes were the incidence of PICS on day 28, 28-day mortality, and in-hospital mortality. A total of 324 well-balanced matched pairs were generated from 1622 patients. The primary outcome did not differ between the antithrombin and control groups (63.9% vs. 68.2%, respectively, *p* = 0.245). However, the incidences of 28-day and in-hospital mortality were significantly lower in the antithrombin group (16.0% vs. 23.5% and 24.4% vs. 35.8%, respectively). Similar results were obtained in a sensitivity analysis using overlap weighting. Antithrombin did not reduce the occurrence of PICS on day 14 in patients with sepsis-induced DIC; however, it was associated with a better mid-term (day 28) prognosis.

## 1. Introduction

Disseminated intravascular coagulation (DIC) is a severe condition with excessive coagulopathy, in which crosstalk between coagulation and inflammation may cause multiple organ dysfunction [1,2]. Thrombus formation and organ failure are prominent, particularly in sepsis-induced DIC [3]. Therefore, the administration of antithrombin (AT), an anti-coagulant agent that may attenuate coagulopathy with the control of inflammation, is effective for and occasionally used to treat DIC in Japan [4]. However, the efficacy of AT for sepsis-induced DIC remains controversial [5]. A reduction in mortality was not examined in detail in a large RCT [6] but was confirmed in a subgroup analysis of DIC [7].

One of the expected effects of AT is the control of hyper-inflammation; therefore, AT may improve some inflammation-related outcomes that are not expressed in mortality. Persistent inflammation following severe conditions is called persistent inflammation, immunosuppression, and catabolism syndrome (PICS) and is one of the most challenging issues in the critical care field [8,9]. Sepsis and DIC are both strong and independent risk factors for the development of PICS [10]. Therefore, interventions with AT to control coagulopathy and hyper-inflammation may prevent PICS and improve the long-term prognosis of sepsis-induced DIC.

Clinical criteria with laboratory data, including C-reactive protein (CRP), albumin, and a lymphocyte count, have been proposed for PICS [8,9]. Among the patients admitted to the ICU, some patients continue to experience prolonged elevation of CRP levels even after 14 days [11]. We previously reported that cut-off values of >2.0 mg/dL for CRP, <3.0 g/dL for albumin, and <800/µL for lymphocyte count on day 14 from admission were appropriate criteria for PICS and may be used to identify patients who develop PICS after critical care [12].

We herein hypothesized that AT prevents the development of PICS and improves the mid-term prognosis of sepsis-induced DIC. To investigate this, we performed a propensity-score-matched analysis of a large database of health-insurance-based admission claims connected with laboratory findings.

## 2. Material and Methods

### 2.1. Data Source

This retrospective cohort study was conducted using the administrative claims database in Japan provided by Medical Data Vision (MDV) Co., Ltd., Tokyo, Japan. This MDV database consists of inpatient administrative data and laboratory test values at Japanese acute care hospitals under the Diagnosis Procedure Combination DPC payment system. The administrative data of 40 million individuals obtained from more than 460 hospitals were included in this database in 2022. Our database in this study contained administrative data on approximately 190,000 patients who were admitted to an intensive care unit (ICU) between April 2008 and September 2021. 

The present study was conducted in accordance with the Declaration of Helsinki and was approved by the Ethics Committee of Hitachi General Hospital (2020-131). The requirement for informed consent was waived due to its retrospective design and the use of anonymized data.

### 2.2. Study Population

We identified adult (≥18 years old) patients admitted to the ICU and diagnosed with sepsis and DIC between April 2008 and September 2021. The diagnostic codes of sepsis and the source of infection were based on a previous study using the DPC database [13]. The diagnosis of DIC used the ICD-10 code of D65. We performed a landmark analysis to account for an immortal time bias with the time point of the third day of hospitalization (“day 2” because the first day of hospitalization was defined as day 0 in the present study). The following patients were excluded from the analysis: (1) women during pregnancy, (2) patients discharged between day 0 and day 2, and (3) patients who died between day 0 and day 2. Patients administered AT during the first three days after hospitalization (day 0, day 1, or day 2) were defined as the AT group, and those who did not have the code of AT during the first three days as the control group. 

### 2.3. Covariates

We extracted the following information as covariates from our database: age, sex, body mass index (categorized as <18.5, 18.5–25.0, 25.0–30.0, or >30.0 kg/m^2^), smoking status (non-smoker or current/ex-smoker), ambulance use, emergency admission, the state of consciousness on admission (alert, confusion, somnolence, or coma categorized using the Japan Coma Scale), comorbidities, laboratory data (white blood cell [WBC], hemoglobin, platelet count, the prothrombin time-international normalized ratio [PT-INR], albumin, aspartate aminotransferase [AST], alanine aminotransferase [ALT], lactate dehydrogenase [LDH], and CRP), the focus of infection, supportive therapies (mechanical ventilation, extracorporeal membrane oxygenation, intra-aortic balloon pumping, polymyxin B hemoperfusion, renal replacement therapy [RRT], noradrenaline [NOA], dopamine, and vasopressin), treatments (systemic antibiotics on day 0 to day 2, sivelestat sodium, systemic steroids, and intravenous immunoglobulin), and transfusion therapy (red blood cells, fresh frozen plasma, platelet concentrate, and albumin). Anticoagulant agents (unfractionated heparin, low-molecular-weight heparin, recombinant thrombomodulin [rTM], gabexate mesilate/nafamostat mesylate, and ulinastatin) were also included as covariates. The updated Charlson Comorbidity Index was calculated as the status of comorbidities using ICD-10 codes [14]. Laboratory examinations as covariates used the worst values obtained during the first three days of admission: the highest values for WBC, PT-INR, AST, ALT, LDH, and CRP, and the lowest values for hemoglobin, the platelet count, and albumin. The statuses of supportive therapies, treatments, and transfusion therapy were assessed using information collected within the first three days of admission. 

### 2.4. Outcomes 

The primary outcome of interest was a composite of the incidence of PICS on day 14 or 14-day mortality. PICS clinical criteria were proposed as prolonged hospitalization > 14 days with CRP > 0.15 mg/dL, total lymphocyte count < 800/μL, and albumin < 3.0 gm/dL; however, there was no basis for cut-off value of each biomarker [8,9]. Our previous study to explore appropriate cut-off values using machine-learning approaches showed the optimal cut-off of each biomarker for PICS as follows [12]: CRP > 2.0 mg/dL, albumin < 3.0 g/dL, and lymphocyte count < 800/μL on day 14. We defined the incidence of PICS when a patient satisfied ≥2 points of these criteria. The date of these laboratory data was referred to the nearest day to day 14 within days 11–17. If laboratory data were not obtained on day 14, but were collected on days 13 and 15, those on day 15 were used in analyses. Patients who were still hospitalized but had not undergone a laboratory test between day 11 and day 17 were not considered to have PICS; therefore, we did not apply the multiple imputation method for the missing values of CRP, albumin, and lymphocyte count on day 14. Secondary outcomes were a composite of 28-day mortality or the incidence of PICS on day 28, the Barthel index at discharge, hospital days, and in-hospital mortality. When calculating the Barthel index at discharge, patients who died during hospitalization were considered to have a score of 0 [15].

### 2.5. Statistical Analysis

The multiple imputation method (20 sets) was used to account for missing values of covariates. We performed a propensity-score-matched analysis to compare outcomes between the AT and control groups based on the propensity scores for each patient [16,17]. A generalized linear regression model with logistic regression using all covariates was employed to estimate the propensity score for receiving AT. The *C*-statistic was calculated to evaluate the goodness of fit. One-to-one nearest-neighbor matching without replacement was performed using a caliper width set at 20% of the standard deviation for propensity scores. Differences in covariates between the AT and control groups before and after matching were described using the standardized mean difference (SMD). SMDs < 0.100 were considered to denote a negligible imbalance between the AT and control groups. After matching, risk differences and the 95% confidence intervals (Cis) of each binomial outcome, such as the incidence of PICS, were calculated, followed by the null hypothesis. Continuous outcomes (the Barthel index at discharge and hospital days) were compared using the Wilcoxon rank-sum test. 

We performed a sensitivity analysis to confirm the robustness of our results using a propensity score weighting method. Since variables were not well balanced using the inverse probability weighting method (data not shown), we applied an overlap weighting analysis [18,19,20]. Overlap weighting is a propensity score weighting method that emphasizes the target population with the most overlap in the observed characteristics between a treatment group and control group. In this method, patients in the treated (AT) group were weighted by the probability of not receiving AT (1–propensity score), while those in the untreated (control) group were weighted by the probability of receiving AT (propensity score). Truncation is not required, since weights are constrained to ranges between 0 and 1 and extreme weights are impossible. Moreover, when the propensity score is estimated by a logistic regression, overlap weighting achieves an exact balance on the mean of every measured covariate. All *p*-values were two-tailed; *p*-values < 0.05 were considered to be significant. All statistical analyses were performed using R (version 4.2.1, R Foundation for Statistical Computing, Vienna, Austria). The “mice”, the “MatchIt”, and the “PSweight” packages were used for the multiple imputation method, propensity score matching, and propensity score weighting, respectively.

## 3. Results

**Figure 1:**
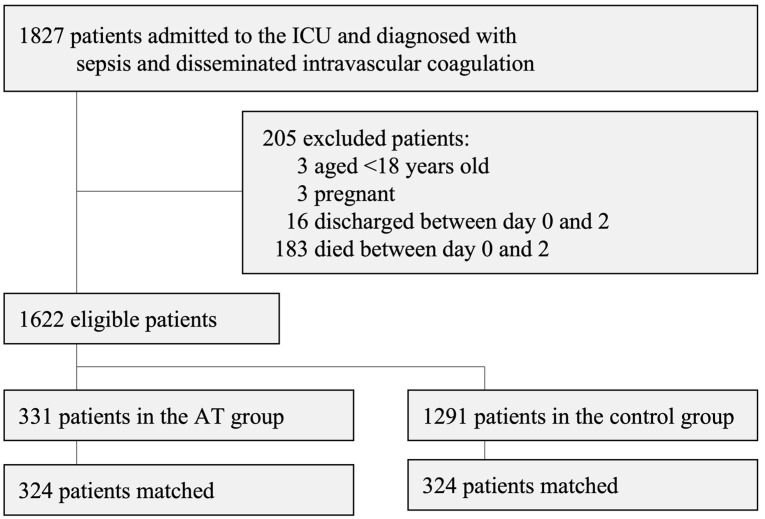
Patient flow chart.

A total of 1827 patients were enrolled in the present study. Following the exclusion of 205 patients, 1622 (331 in the AT group and 1291 in the control group) were included in the propensity score analysis (Figure 1).

Table S1 shows the patient characteristics and the percentages of missing values for each variable in the AT group and the control group. Patient characteristics after applying the multiple imputation method are shown in Table 1. Before matching, patients in the AT group were more likely to be severely ill (a higher percentage receiving mechanical ventilation, RRT, NOA, vasopressin, and transfusion therapy) and to have more severe dysfunctions in the anticoagulant system (a lower platelet count and higher PT-INR). Among 1622 patients, 552 (24.0%) had a diagnosis of abdominal infection, 187 (11.5%) of respiratory infection, and 70 (4.3%) of urogenital infection. rTM was administered to 192 (58.0%) patients in the AT group and 554 (42.9%) in the control group. The *C*-statistic (95%CI) for predicting the administration of AT was 0.74 (0.72–0.77). Propensity score matching generated 324 matched pairs, and the patient characteristics in two groups were well balanced across all covariates (SMDs of all covariates < 0.100). The distributions of propensity scores in the AT and control groups before and after matching are shown in Figure S1. The median (IQR) platelet count of 324 patients in the AT group after matching was 54 (27–89) × 10^9^/L, and 181/324 (55.9%) received mechanical ventilation while 239/324 (73.8%) were administered NOA. In the AT group, 290 (89.5%) patients were administered a dose of 1500 IU/day of AT. The median (IQR) duration of AT administration was 3 (2–3) days.

The results of primary and secondary outcomes before and after matching are shown in Table 2. In the matched cohort, the incidence of a composite of the incidence of PICS on day 14 or 14-day mortality did not significantly differ between the AT and control groups (207/324 [63.9%] vs. 221/324 [68.2%]; risk difference [95%CI], −4.3% [−11.6% to 3.0%]; *p* = 0.245). The incidence of a composite of PICS on day 28 or 28-day mortality was lower in the AT group than in the control group (45.4% vs. 54.0%, respectively; risk difference [95%CI], −8.6% [−16.3% to −1.0%]; *p* = 0.027). Lower risks of 28-day mortality and in-hospital mortality were associated with the administration of AT. No significant difference was observed in hospital days between the two groups. Changes in laboratory data (platelet count, albumin, lymphocyte count, and CRP) from admission to day 28 in the matched cohort are shown in Figure S2. No significant differences were observed in these values on day 0, 7, 14, 21, or 28 between the AT and control groups.

The sensitivity analysis using overlap weighting showed similar results to the main analysis (Table 3). The incidence of the primary outcome did not correlate with the administration of AT (difference [95%CI], −2.8% [−8.6% to 3.0%]), while 28-day mortality and in-hospital mortality were significantly lower in the AT group than in the control group. 

## 4. Discussion

The effects of AT on the development of PICS and mid-term mortality were analyzed in patients with sepsis-induced DIC. AT did not reduce the occurrence of PICS or mortality on day 14. However, 28-day and in-hospital mortalities were lower in the AT group. Therefore, the effect of AT to prevent the development of PICS based on clinical criteria was not confirmed in the present study.

AT did not have a significant impact on PICS clinical criteria on day 14. An immunodeficiency progression is one of the most problematic issues caused by prolonged compensatory anti-inflammatory response syndrome in PICS [8,9]. Since the trigger for the development of PICS is considered to be excessive inflammation, anti-inflammatory medications or agents to control inflammation are expected to be effective approaches for PICS [21]. However, AT did not reduce the occurrence of PICS clinical criteria on day 14, which was originally set for the definition of PICS [8], or on day 28.

No significant differences were observed in 14-day mortality, whereas mid-term mortality on day 28 and in-hospital mortality were lower in the AT group. Similar findings were reported in a survival analysis of subgroups without unfractionated heparin in a previous RCT [6]. Furthermore, the AT intervention improved cognitive functions and activity levels on day 90 in patients in that RCT [22]. These effects of AT on the long-term prognosis of patients may have been based on the control of persistent inflammation, which is not included in PICS clinical criteria. The main mechanism of action of AT that prevents the development of PICS warrants further study.

Once critically ill patients enter the PICS state, their prognosis may be very poor with immunodeficiency and susceptibility to infection as second hits [9]. Therefore, treatment interventions or ICU care in the acute phase of critical care are needed to inhibit the development of PICS [21,23,24]. The control of coagulopathy or DIC is one of the approaches used to suppress the development of PICS [10,24]. Other than anti-inflammatory medication, the management of anemia may prevent PICS [25]. On the other hand, nutrition therapy and early mobilization have been proposed as necessary supportive therapies for PICS [21,23]. Further analyses of the relationships between a number of interventions and PICS are expected and are now being performed with the MDV database used in the present study.

There are several limitations that need to be addressed. Since this was a large but retrospective database analysis, unknown confounding factors may not have been adjusted for. DIC was diagnosed based on health insurance claims; however, we were unable to examine fibrinogen degeneration products, which is necessary to diagnose DIC with the international criteria [26], in the MDV database. PICS clinical criteria have been proposed [8,12], but may not accurately reflect PICS, because of its complexity. Furthermore, we were unable to assess the long-term prognosis of patients, such as 1-year outcomes. A prospective study to investigate the efficacy of AT for PICS conditions and their prognosis is needed.

## 5. Conclusions

In sepsis-induced DIC, AT did not reduce the occurrence of PICS clinical criteria on day 14. However, AT was associated with a better mid-term (day 28) prognosis.

## Figures and Tables

**Table 1:** Patient characteristics of pre-matched and propensity-score-matched cohorts.

	Pre-Matched Cohort			Matched Cohort		
Variables	AT (*n* = 331)	Control (*n* = 1291)	SMD	AT (*n* = 324)	Control (*n* = 324)	SMD
Age, median (IQR)	77 (68–83)	77 (68–84)	0.068	77 (68–83)	79 (68–84)	0.092
Female, *n* (%)	130 (39.3)	574 (44.5)	0.105	128 (39.5)	129 (39.8)	0.006
Body mass index, *n* (%)						
<18.5	82 (24.8)	254 (19.7)	0.123	77 (23.8)	76 (23.5)	0.007
≥18.5, <25.0	193 (58.3)	756 (58.6)	0.005	189 (58.3)	191 (59.0)	0.013
≥25.0, <30.0	44 (13.3)	212 (16.4)	0.088	46 (14.2)	45 (13.9)	0.009
>30.0	12 (3.6)	69 (5.3)	0.083	12 (3.7)	12 (3.7)	<0.001
Current/ex-smoker, *n* (%)	127 (38.4)	468 (36.3)	0.044	128 (39.5)	122 (37.7)	0.038
Charlson Comorbidity Index, median (IQR)	1 (0–3)	1 (0–3)	0.072	1 (0–2)	1 (0–2)	0.059
Ambulance use, *n* (%)	241 (72.8)	949 (73.5)	0.016	236 (72.8)	230 (71.0)	0.041
Emergent admission, *n* (%)	326 (98.5)	1264 (97.9)	0.044	319 (98.5)	318 (98.1)	0.024
Japan Coma Scale at admission, *n* (%)						
Alert	183 (55.3)	687 (53.2)	0.042	180 (55.6)	173 (53.4)	0.043
Confusion	63 (19.0)	338 (26.2)	0.172	63 (19.4)	60 (18.5)	0.024
Somnolence	42 (12.7)	145 (11.2)	0.045	42 (13.0)	48 (14.8)	0.054
Coma	42 (12.7)	121 (9.4)	0.106	39 (12.0)	43 (13.3)	0.037
Laboratory data, median (IQR)						
White blood cells, 10^9^/L	13.8 (9.8–20.5)	14.8 (10.1–22.2)	0.090	13.7 (9.6–19.8)	14.3 (9.8–22.5)	0.078
Hemoglobin, g/dL	9.5 (8.3–10.8)	9.9 (8.3–11.4)	0.170	9.6 (8.3–10.8)	9.4 (8.1–11.0)	0.017
Platelet, 10^9^/L	54 (27–89)	65 (36–114)	0.304	54 (28–86)	53 (28–84)	0.078
Prothrombin time, INR	1.55 (1.34–1.85)	1.44 (1.24–1.73)	0.151	1.55 (1.34–1.89)	1.54 (1.31–1.85)	0.037
Albumin, g/dL	2.1 (1.8–2.4)	2.2 (1.8–2.5)	0.249	2.1 (1.8–2.4)	2.0 (1.7–2.4)	0.031
Aspartate aminotransferase, IU/L	100 (46–296)	77 (36–223)	0.039	92 (45–274)	99 (45–322)	0.046
Alanine aminotransferase, IU/L	46 (21–148)	42 (20–119)	0.029	48 (22–142)	50 (21–151)	0.046
Lactate dehydrogenase, IU/L	362 (252–557)	342 (249–549)	0.060	362 (258–557)	385 (268–623)	0.042
C-reactive protein, mg/dL	25.7 (17.8–31.7)	23.2 (14.7–30.2)	0.157	25.7 (17.9–31.4)	24.8 (17.4–30.4)	0.011
Focus of infection, *n* (%)						
Abdominal	132 (39.9)	420 (32.5)	0.153	128 (39.5)	119 (36.7)	0.057
Blood	2 (0.6)	10 (0.8)	0.021	2 (0.6)	3 (0.9)	0.035
Bone and soft tissue	15 (4.5)	45 (3.5)	0.053	15 (4.6)	14 (4.3)	0.015
Cardiovascular	6 (1.8)	33 (2.6)	0.051	6 (1.9)	8 (2.5)	0.042
Central nervous system	1 (0.3)	22 (1.7)	0.141	1 (0.3)	1 (0.3)	<0.001
Respiratory	31 (9.4)	156 (12.1)	0.088	31 (9.6)	33 (10.2)	0.021
Urogenital	8 (2.4)	62 (4.8)	0.128	8 (2.5)	11 (3.4)	0.055
Others	200 (60.4)	730 (56.5)	0.079	195 (60.2)	202 (62.3)	0.044
Supportive therapies, *n* (%)						
Mechanical ventilation	188 (56.8)	514 (39.8)	0.345	181 (55.9)	181 (55.9)	<0.001
Extracorporeal membrane oxygenation	8 (2.4)	22 (1.7)	0.050	8 (2.5)	4 (1.2)	0.092
Intra-aortic balloon pumping	3 (0.9)	12 (0.9)	0.002	3 (0.9)	1 (0.3)	0.079
Polymyxin B hemoperfusion	54 (16.3)	120 (9.3)	0.423	53 (16.4)	58 (17.9)	0.041
Renal replacement therapy	129 (39.0)	326 (25.3)	0.297	124 (38.3)	125 (38.6)	0.006
Noradrenaline	246 (74.3)	755 (58.5)	0.340	239 (73.8)	247 (76.2)	0.057
Dopamine	78 (23.6)	305 (23.6)	0.001	78 (24.1)	77 (23.8)	0.007
Vasopressin	72 (21.8)	144 (11.2)	0.289	68 (21.0)	67 (20.7)	0.008
Treatments, *n* (%)						
Antibiotics on day 0 to day 2	260 (78.5)	934 (72.3)	0.144	253 (78.1)	257 (79.3)	0.030
Recombinant thrombomodulin	192 (58.0)	554 (42.9)	0.305	189 (58.3)	192 (59.3)	0.019
Unfractionated heparin	281 (84.9)	910 (70.5)	0.351	274 (84.6)	270 (83.3)	0.034
Low-molecular-weight heparin	10 (3.0)	29 (2.2)	0.048	10 (3.1)	10 (3.1)	<0.001
Gabexate mesilate/nafamostat mesilate	187 (56.5)	463 (35.9)	0.423	180 (55.6)	182 (56.2)	0.012
Sivelestat sodium	56 (16.9)	105 (8.1)	0.268	52 (16.0)	55 (17.0)	0.025
Systemic steroids	122 (36.9)	391 (30.3)	0.139	119 (36.7)	117 (36.1)	0.013
Ulinastatin	34 (10.3)	117 (9.1)	0.041	34 (10.5)	35 (10.8)	0.010
Intravenous immunoglobulin	111 (33.5)	221 (17.1)	0.384	108 (33.3)	108 (33.3)	<0.001
Transfusion therapy, *n* (%)						
Red blood cells	118 (35.6)	336 (26.0)	0.210	116 (35.8)	114 (35.2)	0.013
Fresh frozen plasma	114 (34.4)	259 (20.1)	0.327	110 (34.0)	113 (34.9)	0.019
Platelet concentrate	76 (23.0)	184 (14.3)	0.225	73 (22.5)	78 (24.1)	0.037
Albumin	206 (62.2)	474 (36.7)	0.528	199 (61.4)	195 (60.2)	0.025

AT, antithrombin; SMD, standardized mean difference; IQR, interquartile range; INR, international normalized ratio.

**Table 2:** Outcomes in the pre-matched cohort and propensity-score-matched cohort.

	Pre-Matched Cohort		**Matched Cohort**			
Outcomes	AT (*n* = 331)	Control (*n* = 1291)	AT (*n* = 324)	Control (*n* = 324)	Absolute Risk Difference ^†^	*p* Value
Primary outcome						
PICS or mortality on day 14, *n* (%)	212 (64.0)	770 (59.6)	207 (63.9)	221 (68.2)	−4.3 (−11.6 to 3.0)	0.245
PICS on day 14, *n* (%)	178 (53.8)	633 (49.0)	174 (53.7)	179 (55.2)	−1.5 (−9.2 to 6.1)	0.693
14-day mortality, *n* (%)	43 (13.0)	164 (12.7)	40 (12.3)	51 (15.7)	−3.4 (−8.7 to 1.9)	0.213
Secondary outcomes						
PICS or mortality on day 28, *n* (%)	152 (45.9)	574 (44.5)	147 (45.4)	175 (54.0)	−8.6 (−16.3 to −1.0)	0.027
PICS on day 28, *n* (%)	98 (29.6)	322 (24.9)	96 (29.6)	102 (31.5)	−1.9 (−8.9 to 5.2)	0.609
28-day mortality, *n* (%)	55 (16.6)	265 (20.5)	52 (16.0)	76 (23.5)	−7.4 (−13.5 to −1.3)	0.017
The Barthel index at discharge ^‡^, median (IQR)	15 (0–100)	10 (0–95)	15 (0–100)	0 (0–85)	–	0.005
Hospital days, median (IQR)	37 (18–59)	28 (16–52)	37 (18–59)	30 (16–55)	–	0.090
In-hospital mortality, *n* (%)	83 (25.1)	407 (31.5)	79 (24.4)	116 (35.8)	−11.4 (−18.4 to −4.4)	0.001

AT, antithrombin; PICS, persistent inflammation, immunosuppression, and catabolism syndrome; IQR, interquartile range. ^†^ Absolute risk differences were presented as percentages with 95% confidence intervals. ^‡^ The scores of patients who died during hospitalization were analyzed as zero.

**Table 3:** Estimated absolute risk differences between the antithrombin group and control group using the overlap weighting method.

Outcomes	Absolute Risk Difference ^†^	*p* Value
Primary outcome		
PICS or mortality on day 14	−3.1 (−9.3 to 3.2)	0.335
PICS on day 14	−1.1 (−7.5 to 5.4)	0.747
14-day mortality	−2.3 (−6.6 to 2.0)	0.295
Secondary outcomes		
PICS or mortality on day 28	−4.9 (−11.3 to 1.5)	0.134
PICS on day 28	1.2 (−4.7 to 7.0)	0.695
28-day mortality	−6.6 (−11.5 to −1.7)	0.008
In-hospital mortality	−9.5 (−15.2 to −3.8)	0.001

PICS, persistent inflammation, immunosuppression. ^†^ Absolute risk differences were presented as percentages with 95% confidence intervals.

## Data Availability

The datasets generated and analyzed during the present study are available from the corresponding author upon reasonable request.

## Supplementary Materials

The following supporting information can be downloaded at: https://www.mdpi.com/article/10.3390/jcm12113822/s1, Table S1. Patient characteristics before applying multiple imputation method and the numbers (percentages) of missing values for each variable. Figure S1. Distributions of propensity scores in the treated (antithrombin) group and control group before and after matching. Figure S2. Changes in laboratory data after admission in the AT group and control group. A, platelet count; B, albu-min; C, lymphocyte count; D, C-reactive protein. AT, antithrombin; IQR, interquartile range.

## Abbreviations

ALT, alanine aminotransferase; AST, aspartate aminotransferase; AT, antithrombin; CI, confidence interval; CRP, C-reactive protein; DIC, disseminated intravascular coagulation; ICU, intensive care unit; IQR, interquartile range; LDH, lactate dehydrogenase; NOA, noradrenaline; PICS, persistent inflammation, immunosuppression, and catabolism syndrome; PT-INR, prothrombin time-international normalized ratio; RRT, renal replacement therapy; rTM, recombinant thrombomodulin; SMD, standardized mean difference; WBC, white blood cell.

## References

[1] Foley J.H., Conway E.M. (2016). Cross Talk Pathways Between Coagulation and Inflammation. Circ. Res..

[2] Gando S., Levi M., Toh C.H. (2016). Disseminated intravascular coagulation. Nat. Rev. Dis. Prim..

[3] Levi M., van der Poll T. (2017). Coagulation and sepsis. Thromb. Res..

[4] Tagami T., Matsui H., Horiguchi H., Fushimi K., Yasunaga H. (2014). Antithrombin and mortality in severe pneumonia patients with sepsis-associated disseminated intravascular coagulation: An observational nationwide study. J. Thromb. Haemost..

[5] Allingstrup M., Wetterslev J., Ravn F.B., Møller A.M., Afshari A. (2016). Antithrombin III for critically ill patients: A systematic review with meta-analysis and trial sequential analysis. Intensive Care Med..

[6] Warren B.L., Eid A., Singer P., Pillay S.S., Carl P., Novak I., Chalupa P., Atherstone A., Pénzes I., Kübler A. (2001). Caring for the critically ill patient. High-dose antithrombin III in severe sepsis: A randomized controlled trial. JAMA.

[7] Kienast J., Juers M., Wiedermann C.J., Hoffmann J.N., Ostermann H., Strauss R., Keinecke H., Warren B.L., Opal S.M. (2006). Treatment effects of high-dose antithrombin without concomitant heparin in patients with severe sepsis with or without disseminated intravascular coagulation. J. Thromb. Haemost..

[8] Gentile L.F., Cuenca A.G., Efron P.A., Ang D.M., Bihorac A., McKinley B.A., Moldawer L.L., Moore F.A. (2012). Persistent inflammation and immunosuppression: A common syndrome and new horizon for surgical intensive care. J. Trauma Acute Care Surg..

[9] Mira J.C., Gentile L.F., Mathias B.J., Efron P.A., Brakenridge S.C., Mohr A.M., Moore F.A., Moldawer L.L. (2017). Sepsis Pathophysiology, Chronic Critical Illness, and Persistent Inflammation-Immunosuppression and Catabolism Syndrome. Crit. Care Med..

[10] Nakamura K., Ogura K., Nakano H., Naraba H., Takahashi Y., Sonoo T., Hashimoto H., Goto T. (2020). Disseminated Intravascular Coagulopathy Is Associated with the Outcome of Persistent Inflammation, Immunosuppression and Catabolism Syndrome. J. Clin. Med..

[11] Nakamura K., Ogura K., Nakano H., Naraba H., Takahashi Y., Sonoo T., Hashimoto H., Morimura N. (2020). C-reactive protein clustering to clarify persistent inflammation, immunosuppression and catabolism syndrome. Intensive Care Med..

[12] Nakamura K., Ogura K., Ohbe H., Goto T. (2022). Clinical Criteria for Persistent Inflammation, Immunosuppression, and Catabolism Syndrome: An Exploratory Analysis of Optimal Cut-Off Values for Biomarkers. J. Clin. Med..

[13] Tonai M., Shiraishi A., Karumai T., Endo A., Kobayashi H., Fushimi K., Hayashi Y. (2022). Hospital-onset sepsis and community-onset sepsis in critical care units in Japan: A retrospective cohort study based on a Japanese administrative claims database. Crit. Care..

[14] Quan H., Li B., Couris C.M., Fushimi K., Graham P., Hider P., Januel J.-M., Sundararajan V. (2011). Updating and validating the Charlson comorbidity index and score for risk adjustment in hospital discharge abstracts using data from 6 countries. Am. J. Epidemiol..

[15] Schweickert W.D., Pohlman M.C., Pohlman A.S., Nigos C., Pawlik A.J., Esbrook C.L., Spears L., Miller M., Franczyk M., Deprizio D. (2009). Early physical and occupational therapy in mechanically ventilated, critically ill patients: A randomised controlled trial. Lancet.

[16] Griswold M.E., Localio A.R., Mulrow C. (2010). Propensity score adjustment with multilevel data: Setting your sites on decreasing selection bias. Ann. Intern. Med..

[17] Rosenbaum P., Rubin D. (1985). Constructing a Control Group Using Multivariate Matched Sampling Methods That Incorporate the Propensity Score. Am. Stat..

[18] Thomas L.E., Li F., Pencina M.J. (2020). Overlap Weighting: A Propensity Score Method That Mimics Attributes of a Randomized Clinical Trial. JAMA.

[19] Li F., Thomas L.E. (2019). Addressing Extreme Propensity Scores via the Overlap Weights. Am. J. Epidemiol..

[20] Desai R.J., Franklin J.M. (2019). Alternative approaches for confounding adjustment in observational studies using weighting based on the propensity score: A primer for practitioners. BMJ.

[21] Efron P.A., Mohr A.M., Bihorac A., Horiguchi H., Hollen M.K., Segal M.S., Baker H.V., Leeuwenburgh C., Moldawer L.L., Moore F.A. (2018). Persistent inflammation, immunosuppression, and catabolism and the development of chronic critical illness after surgery. Surgery.

[22] Rublee D., Opal S.M., Schramm W., Keinecke H.O., Knaub S. (2002). Quality of life effects of antithrombin III in sepsis survivors: Results from the KyberSept trial [ISRCTN22931023]. Crit. Care.

[23] Moore F.A., Phillips S.M., McClain C.J., Patel J.J., Martindale R.G. (2017). Nutrition Support for Persistent Inflammation, Immunosuppression, and Catabolism Syndrome. Nutr. Clin. Pract..

[24] Horiguchi H., Loftus T., Hawkins R.B., Raymond S.L., Stortz J.A., Hollen M.K., Weiss B.P., Miller E.S., Bihorac A., Larson S. (2018). Innate Immunity in the Persistent Inflammation, Immunosuppression, and Catabolism Syndrome and Its Implications for Therapy. Front. Immunol..

[25] Loftus T.J., Mira J.C., Stortz J.A., Ozrazgat-Baslanti T., Ghita G.L., Wang Z., Brumback B.A., Ungaro R.F., Bihorac A., Leeuwenburgh C. (2019). Persistent inflammation and anemia among critically ill septic patients. J. Trauma Acute Care Surg..

[26] Gando S., Saitoh D., Ogura H., Mayumi T., Koseki K., Ikeda T., Ishikura H., Iba T., Ueyama M., Eguchi Y. (2008). Natural history of disseminated intravascular coagulation diagnosed based on the newly established diagnostic criteria for critically ill patients: Results of a multicenter, prospective survey. Crit. Care Med..

