# Supplementary material for: Effects of Antithrombin on Persistent Inflammation, Immunosuppression, and Catabolism Syndrome among Patients with Sepsis-Induced Disseminated Intravascular Coagulation
Source: J Clin Med. 2023 Jun 2;12(11):3822. doi: 10.3390/jcm12113822 (PMC10253515; doi:10.3390/jcm12113822)
Supplement: Supplementary file 1 [file jcm-12-03822-s001.zip › jcm-2388024-supplementary.pdf]

Journal of
Clinical Medicine

Table S1. Patient characteristics before applying multiple imputation method and the numbers (percentages) of missing values for each variable.

Variables	AT (n = 331)	Control (n = 1,291)
Age, median (IQR)	77 (68–83)	77 (68–84)
Female, n (%)	130 (39.3)	574 (44.5)
Body mass index, n (%)		
<18.5	71 (21.5)	233 (18.0)
≥18.5, <25.0	177 (53.5)	675 (52.3)
≥25.0, <30.0	42 (12.7)	196 (15.2)
>30.0	11 (3.3)	57 (4.4)
missing	30 (9.1)	130 (10.1)
Smoking status, n (%)		
Current/ex-smoker	103 (31.1)	376 (29.1)
Never smoker	171 (51.7)	705 (54.6)
missing	57 (17.2)	210 (16.3)
Charlson Comorbidity Index, median (IQR)	1 (0–3)	1 (0–3)
Ambulance use, n (%)	241 (72.8)	949 (73.5)
Emergent admission, n (%)	326 (98.5)	1,264 (97.9)
Japan Coma Scale at admission, n (%)		
Alert	183 (55.3)	687 (53.2)
Confusion	63 (19.0)	338 (26.2)
Somnolence	42 (12.7)	145 (11.2)
Coma	42 (12.7)	121 (9.4)
missing	1 (0.3)	0 (0)
Laboratory data, median (IQR)		
White blood cells, 10^9/L	13.6 (9.8–19.6)	14.8 (10.1–22.4)
missing, n (%)	35 (10.6)	106 (8.2)
Hemoglobin, g/dL	9.6 (8.3–10.8)	9.8 (8.3–11.4)
missing, n (%)	35 (10.6)	106 (8.2)
Platelet, 10^9/L	56 (28–88)	66 (36–114)
missing, n (%)	35 (10.6)	107 (8.3)
Prothrombin time, INR	1.57 (1.35–1.91)	1.44 (1.24–1.75)
missing, n (%)	55 (16.6)	202 (15.6)
Albumin, g/dL	2.1 (1.8–2.4)	2.2 (1.8–2.6)
missing, n (%)	36 (10.9)	130 (10.1)
Aspartate aminotransferase, IU/L	92 (45–292)	77 (35–212)
missing, n (%)	35 (10.6)	106 (8.2)
Alanine aminotransferase, IU/L	49 (22–150)	42 (20–118)
missing, n (%)	35 (10.6)	108 (8.4)
Lactate dehydrogenase, IU/L	359 (258–551)	348 (251–557)
missing, n (%)	52 (15.7)	259 (20.1)
C-reactive protein, mg/dL	24.7 (17.9–31.3)	22.8 (14.8–29.9)
missing, n (%)	38 (11.5)	123 (9.5)
Focus of infection, n (%)		
Abdominal	132 (39.9)	420 (32.5)
Blood	2 (0.6)	10 (0.8)

**Bone and soft tissue**	15 (4.5)	45 (3.5)
**Cardiovascular**	6 (1.8)	33 (2.6)
**Central nervous system**	1 (0.3)	22 (1.7)
**Respiratory**	31 (9.4)	156 (12.1)
**Urogenital**	8 (2.4)	62 (4.8)
**Others**	200 (60.4)	730 (56.5)
**Supportive therapies, n (%)**		
**Mechanical ventilation**	188 (56.8)	514 (39.8)
**Extracorporeal membrane oxygenation**	8 (2.4)	22 (1.7)
**Intra-aortic balloon pumping**	3 (0.9)	12 (0.9)
**Polymyxin B hemoperfusion**	54 (16.3)	120 (9.3)
**Renal replacement therapy**	129 (39.0)	326 (25.3)
**Noradrenaline**	246 (74.3)	755 (58.5)
**Dopamine**	78 (23.6)	305 (23.6)
**Vasopressin**	72 (21.8)	144 (11.2)
**Treatments, n (%)**		
**Antibiotics on day 0 to day 2**	260 (78.5)	934 (72.3)
**Recombinant thrombomodulin**	192 (58.0)	554 (42.9)
**Unfractionated heparin**	281 (84.9)	910 (70.5)
**Low-molecular-weight heparin**	10 (3.0)	29 (2.2)
**Gabexate mesilate/nafamostat mesilate**	187 (56.5)	463 (35.9)
**Sivelestat sodium**	56 (16.9)	105 (8.1)
**Systemic steroids**	122 (36.9)	391 (30.3)
**Ulinastatin**	34 (10.3)	117 (9.1)
**Intravenous immunoglobulin**	111 (33.5)	221 (17.1)
**Transfusion therapy, n (%)**		
**Red blood cells**	118 (35.6)	336 (26.0)
**Fresh frozen plasma**	114 (34.4)	259 (20.1)
**Platelet concentrate**	76 (23.0)	184 (14.3)
**Albumin**	206 (62.2)	474 (36.7)

AT, antithrombin; IQR, interquartile range; INR, international normalized ratio.

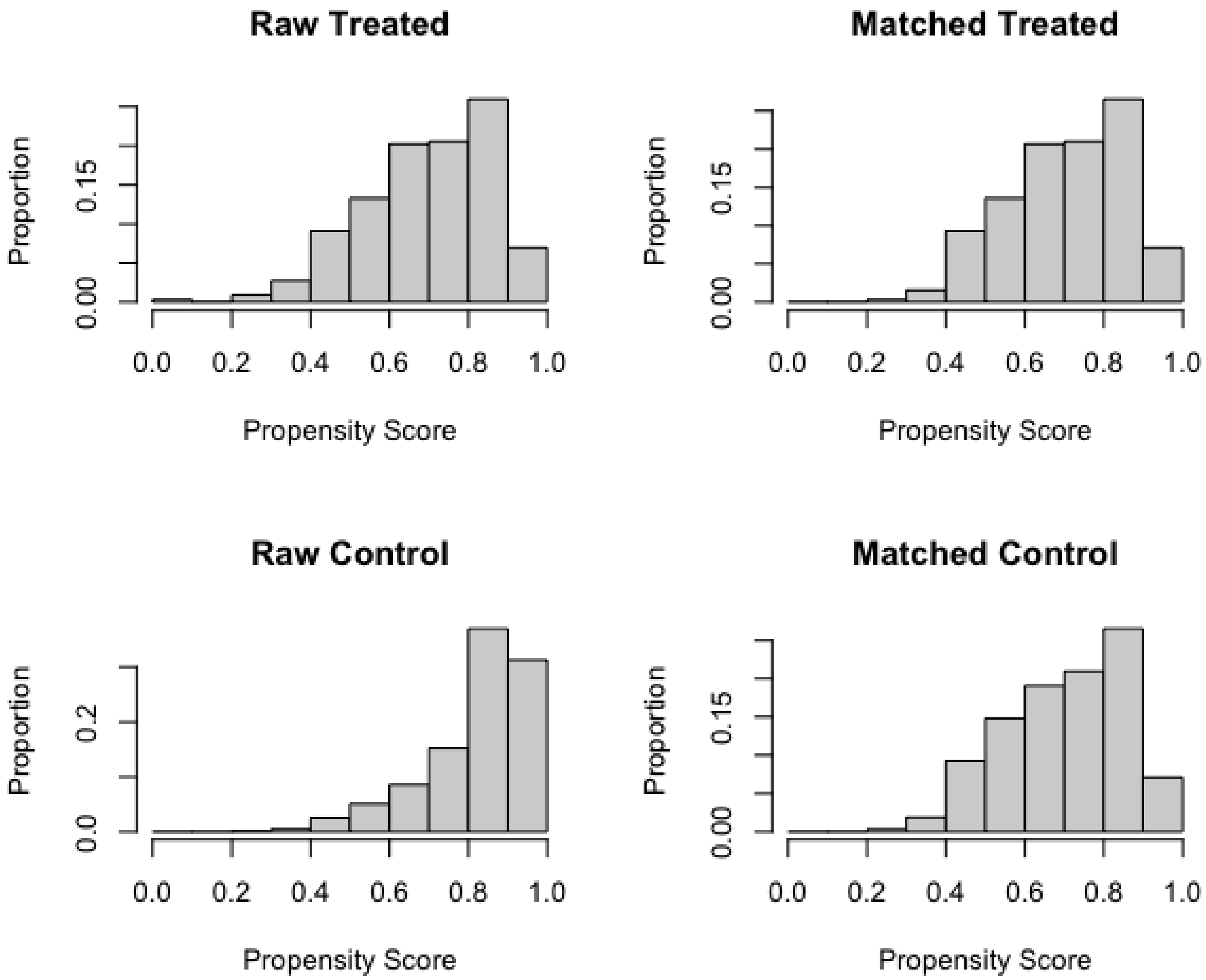

Figure S1. Distributions of propensity scores in the treated (antithrombin) group and control group before and after matching.

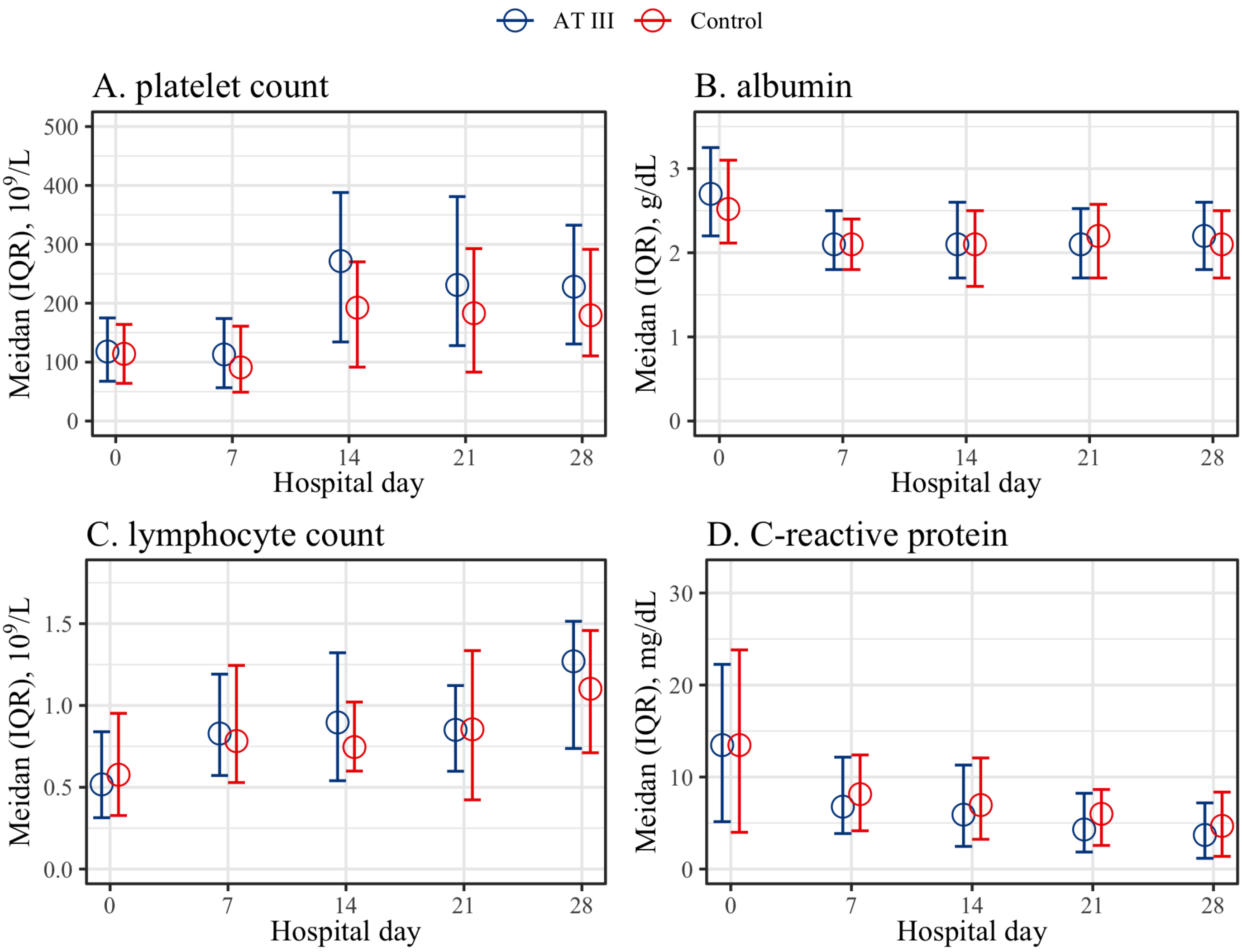

Figure S2. Changes in laboratory data after admission in the AT group and control group. A, platelet count; B, albumin; C, lymphocyte count; D, C-reactive protein. AT, antithrombin; IQR, interquartile range
